# Supplementary material for: The interactome of intact mitochondria by cross-linking mass spectrometry provides evidence for coexisting respiratory supercomplexes
Source: Mol Cell Proteomics. 2017 Dec 8;17(2):216–32. doi: 10.1074/mcp.RA117.000470 (PMC5795388; doi:10.1074/mcp.RA117.000470)
Supplement: Supplemental Data [file supp_RA117.000470_133922_0_supp_23978_fzffwf.zip › spectra_annotation/mito_spectra_annotation/189-1-16-1-8-1.pdf]

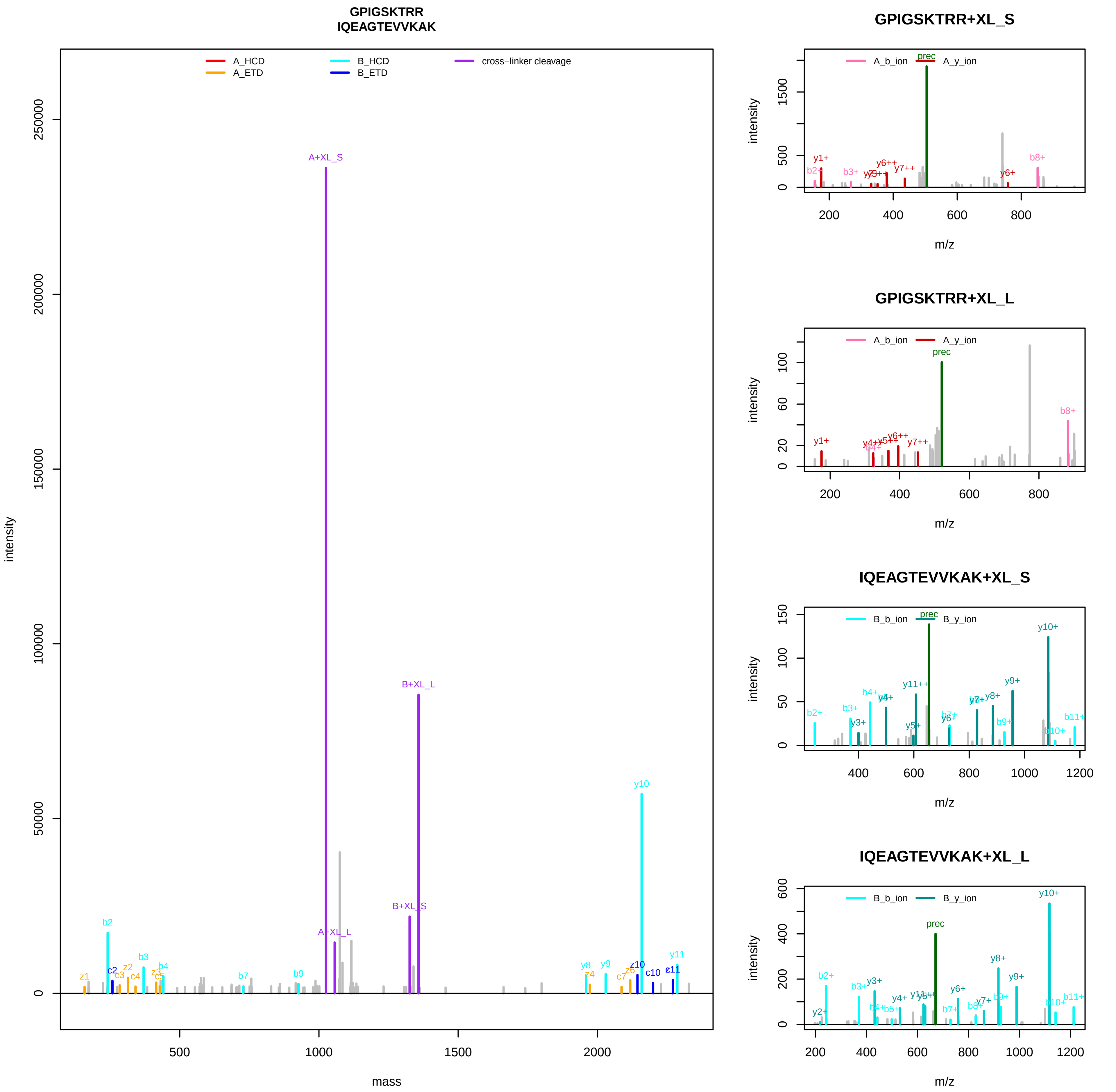
GPIGSKTRR
IQEAGTEVVKAK
A_HCD
A_ETD
B_HCD
B_ETD
cross−linker cleavage
A+XL_S
B+XL_L
B+XL_S
A+XL_L
y10
y11
y8
y9
z4
c7
z6
z10
c10
z11
b2
b3
b4
b7
b9
z1
c2
c3
z2
c4
z3
c5
intensity
mass
0
50000
100000
150000
200000
250000
500
1000
1500
2000
GPIGSKTRR+XL_S
A_b_ion
A_y_ion
prec
y1+
b2+
b3+
y6++
y7++
y6+
b8+
intensity
m/z
GPIGSKTRR+XL_L
A_b_ion
A_y_ion
prec
y1+
y6++
y7++
b8+
intensity
m/z
IQEAGTEVVKAK+XL_S
B_b_ion
B_y_ion
prec
y10+
y11++
y9+
y8+
b4+
y4+
b3+
y3+
b2+
y5+
y6+
b9+
b10+
b11+
intensity
m/z
IQEAGTEVVKAK+XL_L
B_b_ion
B_y_ion
prec
y10+
y8+
y9+
y6+
b2+
b3+
y3+
y4+
y2+
b5+
b7+
b8+
y7+
b9+
b10+
b11+
intensity
m/z
